# Long‐Term Outcomes and Toxicities in Patients With Metastatic Non‐Small Cell Lung Cancer Treated With Immunotherapy Containing Regimens

**DOI:** 10.1002/cnr2.70361

**Published:** 2025-10-08

**Authors:** Meghana Maddula, Lauren J. Brown, Venessa Chin, Bo Gao, Ines Pires Da Silva, Adnan Nagrial

**Affiliations:** ^1^ Crown Princess Mary Cancer Centre, Westmead Hospital Sydney New South Wales Australia; ^2^ University of New South Wales Sydney New South Wales Australia; ^3^ The Kinghorn Cancer Centre, St Vincent's Hospital Sydney New South Wales Australia; ^4^ Medical Oncology, Blacktown Hospital Sydney New South Wales Australia; ^5^ University of Sydney Camperdown New South Wales Australia; ^6^ Westmead Institute for Medical Research Westmead New South Wales Australia; ^7^ The Garvan Institute of Medical Research Sydney New South Wales Australia; ^8^ Melanoma Institute Australia The University of Sydney Sydney New South Wales Australia

**Keywords:** immunotherapy, immunotherapy related adverse events, lung neoplasms, non‐small cell lung carcinoma, real‐world data

## Abstract

**Introduction:**

Immunotherapy is well‐established in treating metastatic non‐small cell lung cancer (mNSCLC); however, data regarding acquired resistance and long‐term outcomes are limited. We examined long‐term outcomes in mNSCLC patients with ongoing treatment response at 2 years (long‐term responders) post‐treatment commencement.

**Methods:**

This multi‐center retrospective study identified mNSCLC patients treated with first‐ or second‐line immunotherapy±chemotherapy. Endpoints included progression‐free survival (PFS) and overall survival (OS), stratified by PD‐L1 tumor proportion score (TPS) (< 50% vs. ≥ 50%), treatment duration, and treatment line.

**Results:**

Of 354 patients, 52 (15%) long‐term responders were identified for analysis. Among them, median age was 68.5 years (28–87); the majority had an ECOG performance status ≤ 1 (81%), high‐PD‐L1 TPS (52%), and adenocarcinoma histopathology (83%). Most (73%) received immunotherapy first‐line. Median treatment duration was 23.5 months (1–80), and 19% prematurely ceased treatment. With a median follow‐up of 39 months from treatment commencement (95% CI 37–49), 15 (29%) patients had progressive disease, and 3‐year PFS was 78%. Oligo‐progression was common (87%), with lung/pleural disease (53%). Most received subsequent treatment (local therapy alone: 53%, systemic therapy alone: 20%, combined: 20%, supportive care: 7%) and achieved disease control (86%). Long‐term toxicities occurred in 44% and were predominantly endocrinopathies (83%) requiring ongoing management. Three‐year OS was 93%. Survival outcomes were unaffected by treatment duration, PD‐L1 TPS, and treatment line.

**Conclusions:**

Long‐term responders showed favorable survival outcomes, with most maintaining disease control with local therapies even after progression. This held true regardless of treatment duration, PD‐L1 TPS, or treatment line. Endocrinopathies were common long‐term toxicities.

## 1 Introduction

Lung cancer remains a leading cause of cancer‐related mortality with historically poor outcomes and 5‐year overall survival (OS) rates as low as 5% [1, 2, 3]. With the advent of anti‐programmed death‐(ligand)1 (PD‐L1) therapies, either alone, or in combination with chemotherapy, survival outcomes have significantly improved [4].

Multiple studies have demonstrated the benefits of immunotherapy in the first‐line treatment setting for metastatic NSCLC across various subgroups [5, 6]. For patients with high PD‐L1 expression (TPS ≥ 50%), immunotherapy alone has shown superior survival outcomes compared to chemotherapy, as seen in KEYNOTE‐024 [5] and IMpower‐110 [6]. Additionally, the combination of chemotherapy and IO has proven effective for patients with lower PD‐L1 expression, though the degree of benefit varies with PD‐L1 levels, as highlighted in KEYNOTE‐042 [7] and KEYNOTE‐189 [8]. Most pivotal studies have limited the duration of IO to 2 years, and in Australia, all first‐line IO treatments are capped at 24 months or 35 cycles of treatment [5, 6, 7, 8]. However, the optimal approach for managing patients beyond 2 years remains unclear and warrants further investigation. In practice, a proportion of patients continue treatment beyond this period, through mechanisms outside standard government reimbursement, including clinical trial participation, compassionate access, or private funding, with continuation decisions made on a case‐by‐case basis.

Although immunotherapy now has a well‐established role in the treatment of metastatic non‐small cell lung cancer (mNSCLC), with 5‐year response rates as high as 32% in the first‐line setting and 13%–25% in the second‐line setting [9, 10, 11], data regarding acquired resistance, particularly beyond first‐line treatment, and long‐term implications are not as well understood as with other immunosensitive cancers [12, 13, 14]. Given this heterogeneity, identifying characteristics of patients with enduring responses is pertinent to guiding ongoing management and surveillance. Evaluation of patterns of progression and subsequent outcomes is also crucial for patient discussions and clinical decision‐making.

Additionally, it is important to recognize the burden of long‐term toxicities associated with immunotherapy, particularly given the limited data on their patterns. Traditional cytotoxic therapy is often associated with acute‐onset adverse events and myelosuppression, whereas immune‐related adverse events (irAE) tend to be diverse, with variable onset‐time and prolonged duration [15, 16]. Toxicities varied between agents, and patient susceptibilities [17], ranging from mild skin toxicities and endocrinopathies, to life‐threatening colitis and pneumonitis [17]. Identifying patterns of long‐term irAE is essential to educate patients, make treatment decisions and effectively manage these complications. As the number of patients treated with immunotherapy increases, along with improving response rates and survivorship, the recognition and management of long‐term toxicities is of increasing concern.

While prior studies including COPILOT [18], an Australian study evaluating patients who completed 2 years of first‐line pembrolizumab monotherapy, and KEYNOTE extension cohorts [5, 7, 8], have reported outcomes in long‐term responders, these have primarily included trial‐eligible patients and often excluded those who discontinued therapy early due to toxicity. Moreover, most have focused on single‐line treatment cohorts without detailed assessment of subsequent progression patterns. In contrast, our study provides additional real‐world insight by examining outcomes in both first‐ and second‐line patients, incorporating a range of immunotherapy‐containing regimens reflective of the current treatment landscape, including those who ceased therapy early, and specifically characterizes patterns of progression with a focus on oligoprogression and its management.

In this study, we aimed to examine long‐term outcomes and toxicities in patients with metastatic NSCLC who showed ongoing disease response to an immunotherapy‐containing regimen in either the first‐ or second‐line setting. We analyzed patterns of disease progression, long‐term toxicities, and the incidence of long‐term immune‐related adverse events (irAE) in patients defined as “long‐term responders”, those without disease recurrence or progression at 2 years post‐treatment commencement.

## 2 Methods and Materials

### 2.1 Study Design and Participants

This retrospective, multi‐center, observational study was conducted across three metropolitan cancer centers in New South Wales, Australia: Westmead Hospital, Blacktown Hospital, and St Vincent's Hospital Sydney. Eligible patients were aged 18 years or older with mNSCLC who received at least one cycle of first‐ or second‐line immunotherapy containing regimens between June 1, 2015 and March 31, 2022. Immunotherapy containing regimens included immune checkpoint inhibitor monotherapy (anti‐PD‐1/PD‐L1/CTLA4) or combination regimens with chemotherapy, consistent with standard of care or clinical trial protocols during the study period. Those with actionable driver mutations were excluded from the study. Patients with ongoing treatment response at 2 years post‐treatment commencement were identified as long‐term responders for further analysis within this study.

This study protocol was approved through the AURORA (Australian Registry and Biobank of Thoracic Cancers) protocol, which is coordinated through the Peter MacCallum Cancer Center and approved by the Peter MacCallum Cancer Center Human Research Ethics Committee (HREC/17/PMCC/42). This approval covers Westmead and Blacktown Hospital sites. Additional approval for St Vincent's Hospital site was obtained from the St Vincent's Hospital Human Research Ethics Committee (HREC/2023/ETH00772). This study was conducted from a start date of March 2022 to data cut‐off for analysis at May 1, 2024. Given the retrospective nature of this study, a waiver of consent was obtained.

Clinical data was extracted from the electronic medical record systems. Patient characteristics, including demographics, medical history, and Eastern Cooperative Oncology Group (ECOG) performance status, were examined. Data on disease characteristics, such as histopathology, mutation status, PD‐L1 tumor proportion score (TPS), and sites of metastases, were also collected. Treatment characteristics, including line of treatment, type, duration, and reason for cessation, were recorded, along with patterns of response, including best response, progression, and management. Oligoprogression was defined as progression in fewer than three metastatic sites, limited to one or two organ sites. This definition was based on the original concept of oligometastatic disease, which was defined as limited to a single or few number of organs, as proposed by Hellman and Weichselbaum [19], and the more recent ESTRO‐ASTRO consensus document, which classified oligoprogressive disease as new or enlarging oligometastases [20, 21]. Information on prior lines of treatment was not available for analysis in this study.

### 2.2 Outcomes and Analyses

The primary efficacy endpoints included progression‐free survival (PFS) and overall survival (OS). PFS and OS were evaluated from the date of treatment commencement to the date of disease progression as determined by individual investigator assessment, death, or censoring. Patients who were lost to follow‐up had their data censored at the time of the last follow‐up visit. Exploratory multivariate analyses including PD‐L1 status and ECOG performance status were attempted but were limited by small cohort size and event numbers.

Secondary endpoints and outcomes included evaluation of baseline clinical and disease characteristics (demographics, ECOG performance status, histopathology, PD‐L1 TPS, sites of metastases), patterns of progression (site and extent of progression, oligoprogression), objective response rate (ORR), and toxicity outcomes. Objective response rate (ORR) was defined as the sum of complete response and partial response and was determined by the treating investigators according to RECIST v1.1 criteria and documented in electronic medical records. As this was a retrospective real‐world study, no blinded central review was performed. Toxicity assessments focused on long‐term immune‐related adverse events (irAE), defined as adverse events persisting 12 months or more since initial occurrence. For this analysis, irAE were defined as on‐treatment if they occurred during active treatment or within 30 days of the last dose, and post‐cessation if they developed beyond this window. These were assessed for type, severity (using CTCAE v5.0 criteria), management, and chronicity.

Subgroup analyses were conducted based on PD‐L1 TPS (high: ≥ 50% and low: < 50%), treatment completion (defined as completing the prescribed course or a minimum of 24 months vs. early cessation), and line of treatment (first‐ vs. second‐line).

### 2.3 Statistical Analysis

Descriptive analysis of patient, disease, and toxicity characteristics included medians and ranges for continuous variables and proportions for categorical variables. Differences in baseline characteristics between each corresponding pre‐specified subgroup were assessed using the Wilcoxon rank‐sum test for continuous variables and Fisher's exact test for categorical variables.

Efficacy endpoints including PFS and OS were evaluated in the overall cohort and stratified by pre‐specified subgroups as outlined above.

PFS and OS survival curves were created using the Kaplan–Meier method, and the Log‐rank test was used to test the difference between each pre‐specified subgroup. Hazard ratios and associated 95% confidence intervals were calculated using a Cox proportional hazards model.

All statistical analyses were performed using *R* (version 4.2.2) and SPSS (version 26).

## 3 Results

### 3.1 Baseline Characteristics

Of 354 patients who received immunotherapy, 52 (15%) long‐term responders were identified. Fifty‐two patients with metastatic NSCLC, treated with an anti‐PD‐L1 agent (either Nivolumab or Pembrolizumab) alone or in combination with chemotherapy, were included in the study as long‐term responders, defined as those with ongoing treatment response at 2 years post‐treatment initiation (Table 1). The median age was 68.5 years (range 28–87), 57.7% were male, and 90.4% were current or former smokers. Most patients (92.3%) had an ECOG performance status (PS) of 0–1.

**TABLE 1:** Patient characteristics.

Characteristic	*N* = 52
Age	68.5 (28–87)
Sex	
Female	42.3% (22)
Male	57.7% (30)
ECOG Status	
0	36.5% (19)
1	55.8% (29)
2	7.7% (4)
Smoking Status	
Current	26.9% (14)
Ex‐smoker (> = 5 PYHx)	53.5% (33)
Never	9.6% (5)
Autoimmune disease at baseline	3.85% (2—Psoriasis)
Histopathology	
Adenocarcinoma	82.7% (43)
Squamous	9.6% (5)
Other	7.7% (4)
PD‐L1 TPS	
> = 50%	51.9% (27)
1%–49%	13.5% (7)
< = 1%	15.4% (8)
Not Known	19.2% (10)
Presence of mutation	40.4% (21)
No mutation	53.8% (28)
Not known	5.8% (3)
Line of treatment	
Second	73.1% (38)
First	26.9% (14)
Treatment Type	
Anti‐PD‐LI (Nivo/Pembro)	67.3% (35)
Anti‐PD‐L1 + Chemo (Carbo/Pem)	32.7% (14)
Cycles of treatment (Median)	32.5 cycles (4–171)
Reason for cessation	
Complete	76.9% (40)
Toxicity (9 prematurely ceased)	19.2% (10)
Other reason for premature cessation (functional decline)	1.9% (1)
Ongoing treatment	1.9% (1)
Best Response	
Complete Response	5.8% (3)
Partial Response	78.8% (41)
Stable disease	15.4% (8)

Adenocarcinoma was the predominant histology, observed in 82.7% of patients, followed by squamous cell carcinoma in 9.6%. PD‐L1 expression was high (TPS ≥ 50%) in 51.9% of patients, while PD‐L1 TPS was unknown in 19.2%. The primary metastatic sites included lymph nodes (94.2%) and thoracic sites such as lung nodules and pleural disease (90.4%). Approximately 36.5% of patients had a high metastatic burden, with three or more distinct metastatic sites.

Most patients (73.1%) received immunotherapy in the first‐line setting. In total, 67.3% of patients were treated with an anti‐PD‐L1 agent alone, while 32.7% received concurrent chemotherapy. The median number of treatment cycles was 32.5, with a median treatment duration of 23.5 months. While the majority of patients discontinued therapy by 24 months or 35 cycles, in alignment with standard treatment guidelines, a small proportion (*n* = 12) continued treatment beyond this period, through mechanisms including clinical trial participation, compassionate access, or private funding, determined by treating clinician on a case‐by‐case basis. Treatment completion or ongoing therapy was observed in 78.9% of patients. Patients with high PD‐L1 expression were more likely to receive first‐line therapy (96.3% vs. 73.3%, *p* = 0.03) and were more frequently treated with single‐agent immunotherapy (74.1% vs. 33.3%, *p* = 0.01). The overall response rate (ORR) was 84.6%.

Premature treatment cessation occurred in 19.2% of patients, with 90.0% due to irAE such as pneumonitis, enterocolitis, and hepatitis. One patient ceased therapy due to declining functional status. Among the cohort of patients who prematurely ceased treatment, the median age was 64 years (range 47–82), 7 were male, and all had an ECOG performance status of 0–1. Most (90%) also had a PD‐L1 TPS >= 1%, with 50% having PD‐L1 TPS >=50%. Of these 10 patients, six received immunotherapy alone and four received chemotherapy and immunotherapy. The median treatment duration for those who discontinued prematurely was 17.5 months. Overall, 81.6% of patients achieved a complete or partial response as the best response to treatment.

### 3.2 Progression Free Survival

With a median follow‐up of 39 months from treatment commencement (95% CI, 37–49), 28.9% in the overall cohort had progressive disease at the time of last follow‐up. The proportion of patients with progressive disease was similar when comparing cohorts treated in the first‐line and second‐line settings (29.0% vs. 28.6%, *p* = 0.98). Disease progression was observed in 50% of patients whose best response was stable disease, compared to 26.8% of those with a partial response and none of those who achieved a complete response (*p* = 0.22).

**FIGURE 1:**
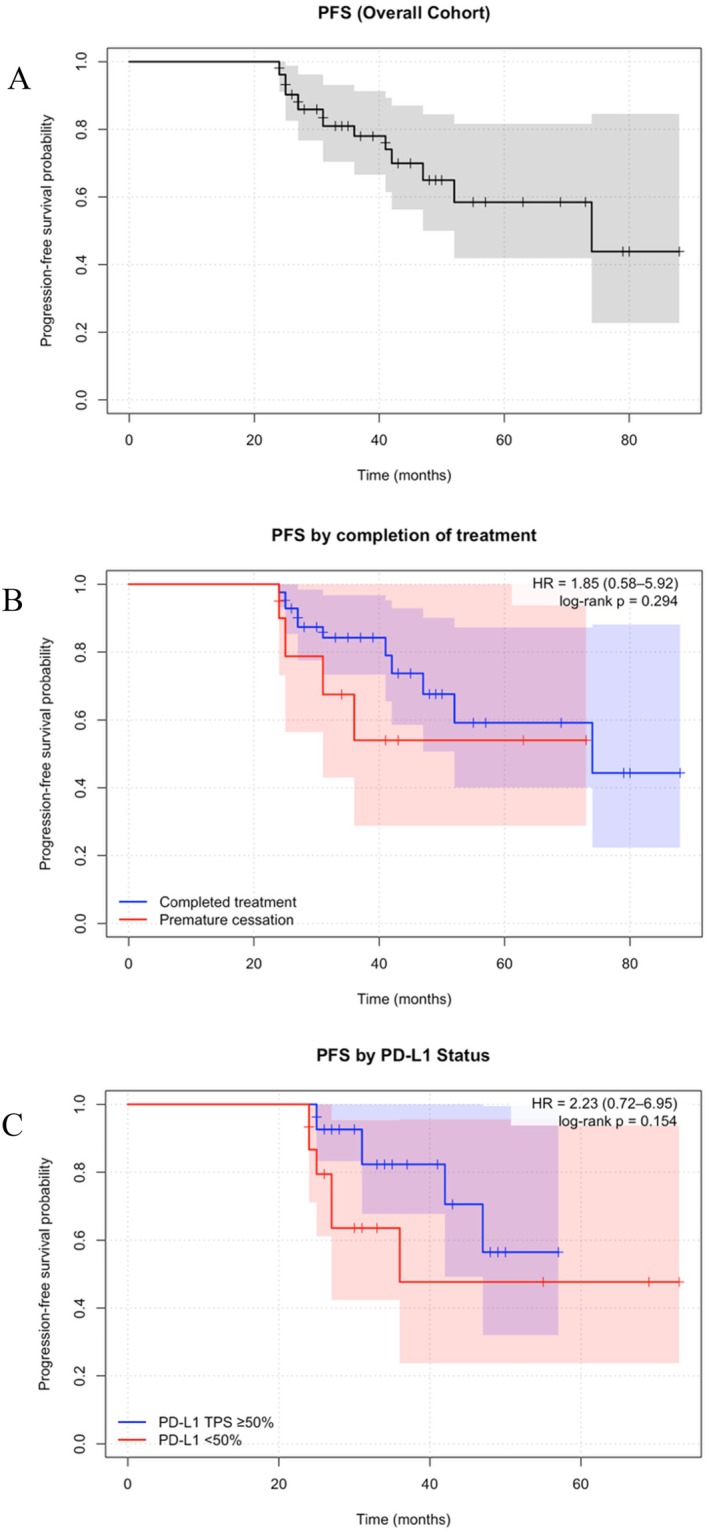
Progression‐free survival (PFS) among long‐term responders with metastatic NSCLC treated with immune checkpoint inhibitors. (A) Kaplan–Meier curve showing PFS for the overall cohort (*n* = 52). Median PFS was 74 months (95% CI, 47 months–not reached), with a 3‐year PFS of 78% (95% CI, 66.6%–91.3%). Shaded regions represent 95% confidence intervals. (B) PFS stratified by treatment completion status. Patients who completed the prescribed treatment course had numerically higher 3‐year PFS compared to those who discontinued early (84.3% vs. 54.0%). The HR for progression in patients with premature cessation was 1.85 (95% CI, 0.58–5.92; log‐rank *p* = 0.294). Shaded regions represent 95% confidence intervals for each group. However these findings were not statistically significant. (C) PFS stratified by PD‐L1 expression. Patients with PD‐L1 TPS ≥ 50% had numerically higher 3‐year PFS compared with those with PD‐L1 TPS < 50% (82.3% vs. 47.7%). The HR for progression in patients with PD‐L1 TPS < 50% was 2.23 (95% CI, 0.72–6.95; log‐rank *p* = 0.154). Shaded regions represent 95% confidence intervals for each group. However these findings were not statistically significant.

In the overall cohort, the 3‐year PFS was 78% (95% CI, 66.6%–91.3%), and median PFS was 74 months (95% CI, 47—NR) (Figure 1). Patients who completed the prescribed treatment course had a numerically higher 3‐year PFS compared to those who did not (84.3% vs. 54.0%, HR 1.85 (95% CI 0.58–5.92), *p* = 0.29) (Figure 1). When stratified by PD‐L1 TPS, the high‐PD‐L1 group (TPS ≥ 50%) had a higher 3‐year PFS in comparison to the lower PD‐L1 TPS group (TPS < 50%) (82.3% vs. 47.7%, HR 2.23 (95% CI 0.72–6.95), *p* = 0.15). The occurrence of toxicity of any grade was not significantly associated with PFS (HR 1.46, 95% CI 0.46–4.6, *p* = 0.52). Exploratory multivariate analyses including PD‐L1 status and ECOG did not identify significant associations, though interpretation was limited by small event numbers.

### 3.3 Characteristics of Progression and Subsequent Treatment

A total of 15 patients who had disease progression. Of these, 87% had oligo‐progression. The most common sites of metastases were thoracic (lung and pleural) (*n* = 8), followed by adrenal (*n* = 3) (Table 2).

**TABLE 2:** Characteristics of disease progression and subsequent treatment.

Features of progression (*N* = 15/52, median follow up 39 months)	
Oligoprogression	86.7% (13)
Systemic/multiple sites	13.3% (2)
Sites of progression	
Respiratory	8
Adrenal	3
Bone	2
Brain	2
Liver	1
Lymph node	1
Large bowel (Colon)	1
Management	
Local therapy only	53.3% (8)
Local therapy + Systemic therapy	20% (3)
Systemic therapy	20% (3)
Best supportive care	6.7% (1)
Best Response to subsequent therapy (*N* = 14)	
Partial response	28.6% (4)
Stable disease	57.1% (8)
Progressive disease	14.3% (2)

In the overall cohort, 53.3% of patients received local therapy alone, primarily radiotherapy (87.5%) and occasionally surgery (12.5%). All of these patients continued on their initial immunotherapy regimen while receiving radiotherapy.

Among patients with oligo‐progression, a higher proportion (66.7%) received local therapy alone, with seven patients receiving radiotherapy and one patient undergoing surgery for intracranial metastasis. Three patients (23%) were treated with a combination of local therapy and systemic therapy, and two patients (15%) with systemic therapy alone.

In the overall cohort, three patients (20%) received subsequent systemic therapy (Carboplatin/Pemetrexed [*N* = 1], Docetaxel [*N* = 1], Clinical trial [*N* = 1]) and one patient received best supportive care (Table 2).

Of the 14 patients who received any subsequent treatment, the disease control rate was 86.0%; 57.1% of patients achieved stable disease, and 28.6% had a partial response to treatment, and only 14.3% had further disease progression by investigator assessment based on RECIST v1.1.

There were no statistically significant differences in baseline characteristics between patients who progressed vs. those that did not; however, there were some notable trends (Table 3). There was a larger proportion of women in the group that had progressive disease compared to the group that did not (60.0% vs. 35.1%, *p* = 0.10). Median age and smoking history were comparable across both groups. The proportion of patients with an ECOG PS of 0 was also higher in the group of patients that did not progress (43.2% vs. 20%, *p* = 0.17).

**TABLE 3:** Characteristics by disease progression.

Characteristic	Progressed (*N* = 15)	Did not progress (*N* = 37)	*P* values
Age	68 (47–83)	70 (28–87)	0.21
Sex			
Female	60% (9)	35.1% (13)	0.10
Male	40% (6)	64.9% (24)	
ECOG Status			
0	20% (3)	43.2% (16)	0.17
1	66.7% (10)	51.4% (19)	
2	33.3% (2)	13.3% (2)	
Smoking Status			
Current	20% (3)	5.4% (2)	0.26
Ex smoker (> = 5 PYHx)	26.7% (4)	27.0% (10)	
Never	53.3% (8)	67.6% (25)	
Autoimmune disease at baseline	0% (0)	5.4% (2)	0.36
Histopathology			
Adenocarcinoma	86.7% (13)	81.1% (30)	0.88
Other	13.3% (2)	18.9% (7)	
PD‐L1 TPS			
> = 50%	40% (6)	56.8% (21)	0.67
> = 1%–50%	20% (3)	10.8% (4)	
< 1%	20% (3)	13.5% (5)	
Not Known	20% (3)	18.9% (7)	
Line of treatment	73.3% (11)	73.0% (27)	0.98
First	26.7% (4)	27.0% (10)	
Second			
Presence of Mutation	60% (9)	32.4% (12)	0.16
Not known	6.7% (1)	5.4% (2)	
Treatment Type	60% (9)	70.3% (26)	0.47
Anti‐PD‐L1 (Nivo/Pembro)	40% (6)	29.7% (11)	
Anti‐PD‐L1 + Chemo (Carbo/Pem)			
Cycles of treatment (Median)	35	32	0.78
Completed/Ongoing treatment	73.3% (11)	97.3% (36)	
Ongoing (*N* = 1)			0.39
Best Response			
Complete Response	0% (0)	8.1% (3)	0.22
Partial Response	73.3% (11)	81.1% (30)	
Stable disease	26.7% (4)	10.8% (4)	

### 3.4 Overall Survival

**FIGURE 2:**
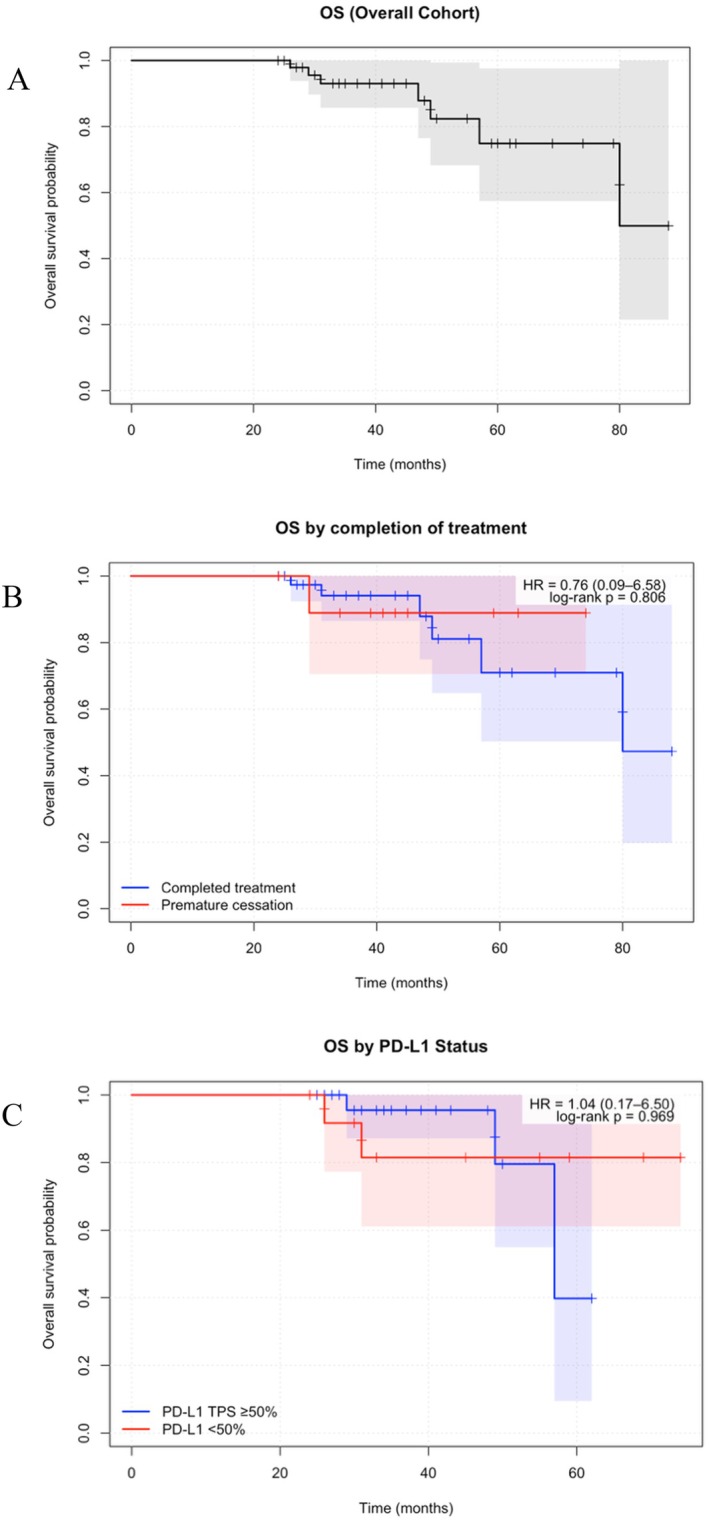
Overall survival (OS) among long‐term responders with metastatic NSCLC treated with immune checkpoint inhibitors. (A) Kaplan–Meier curve showing OS for the overall cohort (*n* = 52). Median OS was 80 months (95% CI, 80–not reached), with a 3‐year OS of 93.0% (95% CI, 85.7%–100%). Shaded regions represent 95% confidence intervals. (B) OS stratified by treatment completion status. Patients who discontinued therapy prematurely had survival outcomes similar to those who completed the full course. The HR for death in patients with premature cessation compared with those completing therapy was 0.76 (95% CI, 0.09–6.58; log‐rank *p* = 0.81). Shaded regions represent 95% confidence intervals for each group. There was no statistically significant difference between groups. (C) OS stratified by PD‐L1 expression. Patients with PD‐L1 TPS ≥ 50% and those with PD‐L1 TPS < 50% demonstrated comparable survival outcomes. The HR for death in patients with PD‐L1 TPS < 50% compared with ≥ 50% was 1.04 (95% CI, 0.17–6.50; log‐rank *p* = 0.97). Shaded regions represent 95% confidence intervals for each group. There was no statistically signficant difference between groups.

At a median follow‐up of 39 months from treatment commencement (95% CI, 37–49), OS was 86.5%, 3‐year OS was 93.0% (85.7%–100%, 95% CI), and median OS was 80 months (95% CI, 80‐NR) (Table 2). When stratified by line of treatment, median OS was not reached in either cohort. There were no statistically significant differences in OS when stratified by PD‐L1 TPS (HR 1.04, 95% CI 0.17–6.50, *p* = 0.97) or treatment completion (HR 0.76, 95% CI 0.09–6.58, *p* = 0.81) (Figure 2). No baseline patient or disease features were associated with OS. The occurrence of toxicity of any grade was not significantly associated with OS (HR 2.87, 95% CI 0.34–23.89, *p* = 0.33). Exploratory multivariate analyses including PD‐L1 status and ECOG did not identify significant associations, though interpretation was limited by small event numbers.

Among patients with oligoprogressive disease (*n* = 13), the small subgroup size limited statistical analysis; however, survival outcomes remained favorable across treatment approaches, with 36‐month survival rates of 86% for local therapy alone and 100% for both combined systemic and local therapy as well as systemic therapy alone.

### 3.5 Toxicity Assessment

The majority (65.4%) of long‐term responders sustained irAE of any grade. All toxicities were on‐treatment toxicities. The most common toxicities were thyroiditis (41.2%), skin toxicity (35.3%), and pneumonitis (17.7%). As evaluated by CTCAE grading, 59.6% of toxicities were grade 1, with 2 instances of grade 4 enterocolitis, and no grade 5 toxicities. The majority (59.6%) of irAE occurred at or later than 6 months post‐initial treatment commencement (Table 4).

**TABLE 4:** Toxicity assessment.

Toxicity event (No. patients = 34/65.4%)	No. of events (*N* = 52)	Grades		Time to toxicity		No. events leading to temporary Cessation	No. events leading to permanent Discontinuation	Immunosuppressive therapy required
		1–2	3–4	< 6 months (47%)	>= 6 months (53%)			
Thyroiditis	14	13	0	9	0	0		0
Skin	12	12	0	2	1	1		1
Pneumonitis	6	4	2	3	4	4		4
Hypophysitis	4	4	0	1	0	0		0
Enterocolitis	8	3	5	1	1	1		1
Hepatitis	3	1	2	1	2	2		2
AI Diabetes	2	2	0	2	0	0		0
Arthritis	2	2	0	1	0	0		0
Nephritis	1	1	0	0	0	0		0

Long‐term toxicities			
Toxicity event	No. of events (N = 52)	Ongoing toxicity (No. patients = 15/44.1%)	Management
Thyroiditis	14	14	All required ongoing hormone replacement.
Skin	12	3	Managed with topical steroids.
Pneumonitis	6	0	
Hypophysitis	4	4	All required ongoing steroid replacement.
Enterocolitis	8	0	
Hepatitis	3	0	
AI Diabetes	2	2	All required ongoing insulin use.
Arthritis	2	1	Required 2 weeks of Leflunomide, and 15 months of Methotrexate.
Nephritis	1	0	

Systemic immunosuppression (steroids or biologic agents) was required for the management of 38.5% of irAE. Pneumonitis and hypophysitis, followed by enterocolitis, were the most common toxicities requiring steroid therapy, with a median prednisone‐equivalent dose of 75 mg, either weaned to a maintenance dose or to cessation over a minimum of 4 weeks. Two patients experienced grade 4 enterocolitis, and 1 patient experienced grade 3 enterocolitis requiring Infliximab (350 mg single dose). One patient experienced grade 2 arthritis requiring Methotrexate (20 mg/week for 2 weeks) and Leflunomide (10 mg/day for 15 months). All skin toxicities required topical steroid therapy alone.

Seven patients required temporary treatment cessation, and 10 patients required permanent treatment discontinuation due to irAE, with nine patients ceasing treatment prior to completion of the prescribed course or 2 years. One patient required premature treatment cessation due to a decline in ECOG performance status. Pneumonitis of any grade was the most common cause for treatment cessation (40.0%). Other adverse events leading to permanent treatment discontinuation included enterocolitis, hepatitis, and arthritis. One patient had both grade 3 pneumonitis and hepatitis. One patient with grade 2 skin toxicity also ceased therapy in the setting of complete metabolic response, and one patient with grade 2 enterocolitis also ceased therapy having completed over 2 years of treatment.

A total of 15 patients (44.1%) had long‐term toxicity, defined as present 12 months post‐treatment commencement. All endocrine irAE resulted in irreversible dysfunction requiring ongoing hormone replacement, with no spontaneous recovery observed. These comprised 83.3% of all long‐term irAE endocrinopathies: thyroiditis (58.3%), hypophysitis (16.7%), and diabetes (8.3%) requiring ongoing hormone replacement. Other ongoing toxicities included skin toxicity (12.5%) and arthritis (4.2%). All skin toxicities were managed with topical therapies alone.

## 4 Discussion

This multi‐center, retrospective study aimed to examine the long‐term outcomes of patients with metastatic NSCLC (mNSCLC) who achieved a sustained response to immunotherapy in real‐world settings. Specifically, we focused on a small cohort of long‐term responders, defined as patients with ongoing response at 2 years post‐treatment initiation. Our analysis addressed several key areas: the durability of response in a subset of real‐world patients, the prevalence of oligoprogression and the effectiveness of local therapies, and the prevalence and implications of long‐term irAE. Our study revealed that approximately 15% of real‐world patients with metastatic NSCLC were classified as long‐term responders. This benefit appeared independent of PD‐L1 expression, treatment line, or early cessation of therapy. This finding suggests a unique subset of patients who derive substantial, long‐lasting benefit from immunotherapy, aligning with observations from key clinical trials [5, 6, 7, 8]. Although our cohort was also largely representative of the NSCLC patient population outside of a trial setting, it had a lower median age compared to the generally reported median age of 70, as well as better ECOG performance status than this broader population [22, 23, 24, 25].

Unlike prior studies that largely evaluated trial‐eligible patients on first‐line monotherapy [5, 11, 17], our study deliberately incorporated a more heterogeneous real‐world population, including both first‐ and second‐line patients, those treated with chemotherapy‐immunotherapy combinations, and patients who discontinued treatment early. This broader inclusion reflects contemporary clinical practice more accurately and provides a unique contribution to the existing evidence base.

The survival outcomes observed in this study are notably superior to historical benchmarks for mNSCLC [1, 2, 3]. By focusing on long‐term responders, we have identified a specific population likely to achieve extended survival, providing insights into the characteristics associated with these outcomes. This benefit was seen across both first‐ and second‐line settings, and across PD‐L1 TPS scores. For context, the KEYNOTE‐024 5‐year update [5] reported a 5‐year OS of 31.9% with pembrolizumab monotherapy in PD‐L1 ≥ 50% patients. Similarly, the pooled 5‐year analysis of CheckMate‐017/−057 [11] demonstrated a 5‐year OS of 13.4% with nivolumab in previously treated patients. This difference reflects both the enrichment of our cohort for long‐term responders and the broader inclusion of chemotherapy‐immunotherapy regimens, patients with variable PD‐L1 expression, and those discontinuing early due to toxicity, all groups not fully captured in trial populations. These contrasts highlight the distinct contribution of our study, emphasizing how durable immunotherapy benefit extends across more heterogeneous, real‐world cohorts.

The median duration of therapy in our study was 23.5 months (range 1–80), with standard treatment recommendations capped at 24 months or 35 cycles under PBS reimbursement. However, unlike KEYNOTE‐024 [5] and CheckMate‐017/057 [11], a small proportion of patients in our cohort continued beyond this period, accessing therapy through clinical trial enrolment, compassionate access programs, or private funding. These decisions were made on a case‐by‐case basis, considering clinical benefit, tolerability, patient preference, and clinician judgment. This context is important when interpreting our findings, as it reflects deviations from standard treatment duration that occur in real‐world practice.

Disease progression occurred in approximately one‐third of long‐term responders, with most cases classified as oligoprogression affecting thoracic sites, a pattern also reported in other studies [26]. Local therapies successfully managed these cases, achieving disease control without the need for changes in systemic therapy. Our study, to our knowledge, is one of the few to describe the management of oligopression in long‐term responders, highlighting the role of local therapies in extending disease control without systemic escalation.

Our study also highlights the relationship between initial response and long‐term outcomes. Among patients with stable disease as their best response, 50% experienced subsequent progression, whereas none of those with a complete response had disease progression. Furthermore, premature treatment cessation due to irAE did not negatively affect rates of progression, PFS, or OS, suggesting durable responses may persist even without continuous therapy. This observation is consistent with CheckMate 017/057 [11], where long‐term survivors were often those achieving deep or durable responses, and with KEYNOTE‐024 [5], where durable benefit was maintained in a subset even after discontinuation. In line with this, survival outcomes were also not significantly different between patients who experienced irAE and those who did not. This analysis provides some reassurance that durable responses are achievable in this unique subgroup of patients, a perspective often underrepresented in clinical trial data. However, as this cohort was restricted to ‘long‐term responders,’ these outcomes may reflect a population with favorable prognosis, and response and survival estimates should be interpreted in this context. This phenomenon, however, has also been observed in other cancers, such as melanoma, where durable responses correlate with improved survival, while primary resistance predicts a poorer prognosis [27, 28].

While immunotherapy has transformed the treatment landscape of metastatic NSCLC, our study highlights the frequency, burden, and long‐term implications of irAE in these patients, which are often under‐reported in clinical trial data. Many patients developed irAE later in the treatment timeline, with a significant proportion occurring 6 months or later from treatment commencement in contrast to acute‐onset toxicities associated with chemotherapy [15, 16, 29]. Within our cohort, we did not observe any new irAE emerging after treatment cessation, however. There was a high prevalence of endocrinopathies requiring ongoing management and hormone replacement, as demonstrated in other studies [30, 31]. A significant proportion of these adverse events persisted for 12 months or longer, highlighting the need for long‐term irAE surveillance as part of survivorship care. Patients often misunderstand the possibility of delayed or prolonged irAE, even after treatment cessation, underscoring the importance of patient education in this regard [15, 16, 29]. Future studies should further examine rates and severity of toxicities, particularly grade ≥ 3 events and their association with survival outcomes.

The COPILOT study [18], also conducted in Australia, provides a relevant comparison to our findings. COPILOT focused on patients completing 2 years of first‐line Pembrolizumab monotherapy, with similar baseline characteristics to our cohort, including median age, ECOG status, and high PD‐L1 expression. Both studies demonstrated favorable survival outcomes, with our median PFS of 47 months closely aligning with COPILOT's 46.1 months and an ORR of 84.6% versus 78.6% in COPILOT [18]. Our higher observed ORR likely reflects both the enrichment of our cohort for long‐term responders and the reliance on investigator‐assessed outcomes, a limitation inherent to retrospective analyses, and the results should therefore be interpreted within this context. Our 3‐year OS was 93%, slightly lower than COPILOT's 98.2%, potentially reflecting differences in therapy duration, treatment line, and regimen. Unlike COPILOT, which also captures outcomes in a trial‐like cohort of patients, our study specifically examined long‐term responders treated with Pembrolizumab or Nivolumab, in both monotherapy and chemotherapy‐immunotherapy regimens, across both first‐ and second‐line settings, and including those who prematurely ceased therapy. The inclusion of patients on second‐line therapy (26.9%), patients who received chemotherapy‐based regimens (40%), and patients who did not complete 24 months of therapy (21.2%) may explain the difference in OS seen between these studies, despite the other similarities between the cohorts. Our findings show that durable response to immunotherapy may extend across treatment regimens and settings, and even in the context of early discontinuation due to irAE, highlighting that long‐term benefit persists in real‐world cohorts.

## 5 Limitations and Considerations

This study has several limitations, including its retrospective design and potential for selection bias. Although conducted across three sites, the cohort size of long‐term responders was modest, reflecting the potential rarity of this population; however, this, accompanied by low event numbers, not only limited statistical power for subgroup analyses and generalisability but also precluded more robust multivariate analyses to adjust for potential confounders. This study has several limitations, including its retrospective design and associated risk of bias. Although conducted across three sites, the number of long‐term responders was modest, reflecting the rarity of this population. The small cohort and low number of events limited statistical power for subgroup analyses, reduced generalisability, and precluded robust multivariate adjustment for potential confounders. By restricting inclusion to patients who remained progression‐free for 2 years, the study inherently selected for individuals with a favorable prognosis, which may have led to overestimation of response and survival outcomes. Disease progression was assessed by treating physicians without central radiologic review, potentially introducing variability in outcome assessment. Finally, reliance on clinical documentation may have incompletely captured late irAEs or progression events, and the study lacked power to identify predictors of long‐term response or toxicity.

Nonetheless, this study offers valuable clinical insights into managing mNSCLC patients who achieve durable responses to immunotherapy in real‐world settings. By characterizing long‐term outcomes, progression patterns, and long‐term irAE, our findings contribute to the growing body of evidence that can guide clinicians in the care of this unique patient population.

## 6 Conclusions and Future Directions

This real‐world cohort of long‐term responders with mNSCLC exhibited favorable survival outcomes across various PD‐L1 expression scores and treatment lines, demonstrating durable response to treatment, even when treatment is prematurely discontinued due to toxicity. The high rate of oligoprogression observed suggests that local therapies offer an effective option for managing limited disease progression, without the need to alter systemic therapy. Furthermore, the high prevalence of long‐term irAE in this cohort emphasizes the need to incorporate extended monitoring and patient education into survivorship care, with clinician vigilance essential for effectively managing these delayed toxicities.

Future research should focus on validating these findings in larger, prospective cohorts, with an emphasis on comprehensive multivariable analyses to adjust for key confounders. This is essential to refine prognostic markers and better define predictors of durable response, which remain incompletely understood. In parallel, exploration of optimal strategies for managing oligoprogression and continued surveillance of long‐term irAE will be increasingly important as the population of long‐term responders grows.

## Author Contributions

**Meghana Maddula:** conceptualisation, Data curation, Formal analysis, Methodology, writing original draft, Writing – review and editing. **Lauren J. Brown:** data curation, supervision, writing – review and editing. **Ines Pires Da Silva:** methodology, writing – review and editing. **Adnan Nagrial:** writing – review and editing. **Venessa Chin:** writing – review and editing.

## Ethics Statement

The study was conducted according to the guidelines of the Declaration of Helsinki. This study protocol was approved through the AURORA protocol approved by the Peter MacCallum Cancer Center Ethics Committee (HREC/17/PMCC/42) and St Vincent's Hospital Human Research Ethics Committee (HREC/2023/ETH00772).

## Consent

The study was conducted according to the guidelines of the Declaration of Helsinki. This study protocol was approved through the AURORA protocol approved by the Peter MacCallum Cancer Centre Ethics Committee (HREC/17/PMCC/42) and St Vincent's. Hospital Human Research Ethics Committee (HREC/2023/ETH00772). Given the retrospective nature of this study, a waiver of consent was obtained.

## Conflicts of Interest

The authors declare no conflicts of interest.

## Data Availability

The data that support the findings of this study are available on request from the corresponding author. The data are not publicly available due to privacy or ethical restrictions.
